# Supplementary material for: Ageing impairs the regenerative capacity of regulatory T cells in mouse central nervous system remyelination
Source: Nat Commun. 2024 Mar 11;15:1870. doi: 10.1038/s41467-024-45742-w (PMC10928230; doi:10.1038/s41467-024-45742-w)
Supplement: Supplementary file 1 — Supplementary Information [file 41467_2024_45742_MOESM1_ESM.pdf]

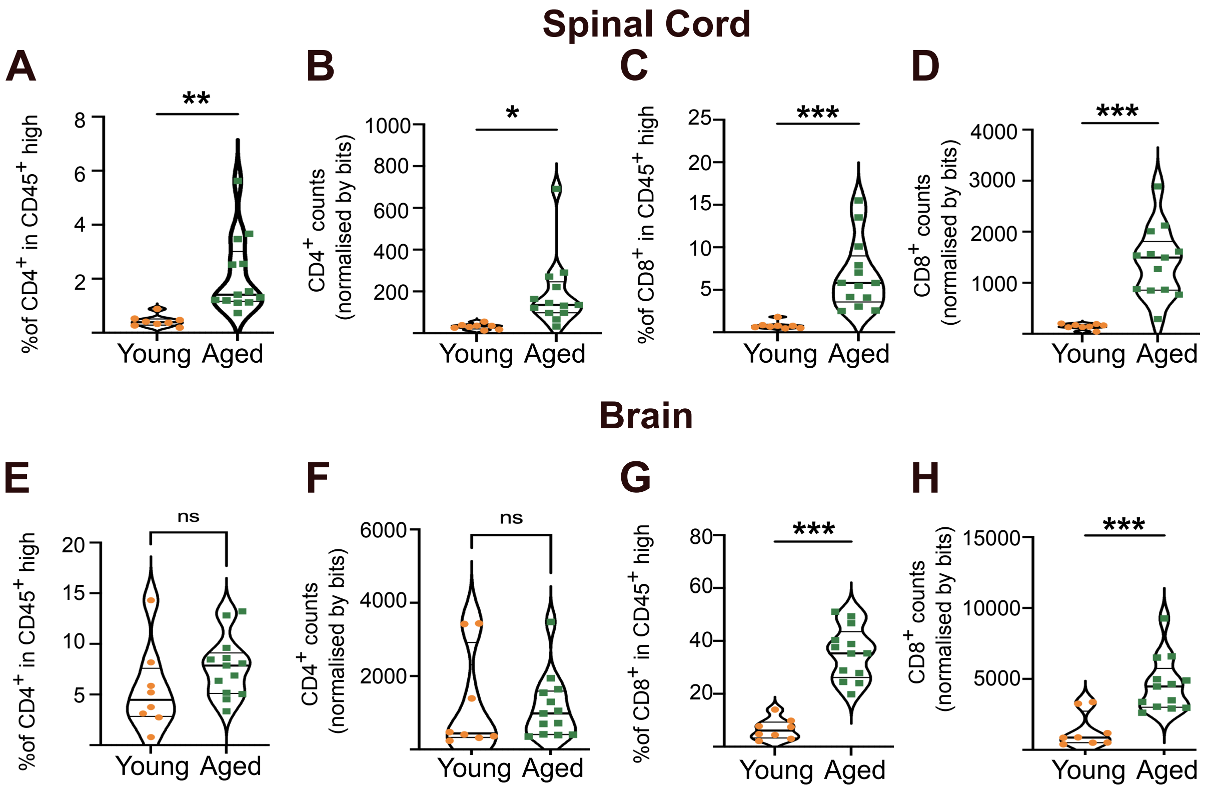
Spinal Cord
A
B
C
D
%of CD4+ in CD45+ high
CD4+ counts (normalised by bits)
%of CD8+ in CD45+ high
CD8+ counts (normalised by bits)
**
*
***
***
Young
Aged
Brain
E
F
G
H
ns
ns
***
***

Sup. Fig. 1

Supplementary Figure 1: CD4$^+$ and CD8$^+$ T cells are increased in the aged CNS. A Violin plot-based quantification showing the proportion of CD4$^+$ T cells out of CD45$^+$ high cells in the spinal cord of healthy young (n=8 mice) and aged (n=13 mice) mice (unpaired two-tailed Student's t test after *arcsin* conversion, t=3.314, **P=0.0036). **B** Violin plot-based quantification showing the count of total CD4$^+$ T cells in the spinal cord of healthy young (n=8 mice) and aged (n=13 mice) mice (unpaired two-tailed Student's t test, t=2.626, *P=0.0166). **C** Violin plot-based quantification showing the proportion of CD8$^+$ T cells out of CD45$^+$ high cells in the spinal cord of healthy young (n=8 mice) and aged (n=13 mice) mice (unpaired two-tailed Student's t test after *arcsin* conversion, t=4.029, ***P=0.0007). **D** Violin plot-based quantification showing the total CD8$^+$ T cells in the spinal cord of healthy young (n=8 mice) and aged (n=13 mice) mice (unpaired two-tailed Student's t test after, t=5.053, ***P<0.0001). **E** Violin plot-based quantification showing the proportion of CD4$^+$ out of CD45$^+$ high cells in the brain of healthy young (n=8 mice) and aged (n=13 mice) mice (unpaired two-tailed Student's t test after *arcsin* conversion, t=1.399, P=0.1779). **F** Violin plot-based quantification showing the total CD4$^+$ T cell count in the brain of healthy young (n=8 mice) and aged (n=13 mice) mice (unpaired two-tailed Student's t test, t=0.2195, P=0.8286). **G** Violin plot-based quantification showing the proportion of CD8$^+$ out of CD45$^+$ high cells in the brain of healthy young (n=8 mice) and aged (n=13 mice) mice (unpaired two-tailed Student's t test after *arcsin* conversion, t=7.425, ***P<0.0001). **H** Violin plot-based quantification showing the total CD8$^+$ T cells in the brain of healthy young (n=8 mice) and aged (n=13 mice) mice (unpaired two-tailed Student's t test, t=4.232, ***P=0.0005). Source data are provided as a Source Data File.

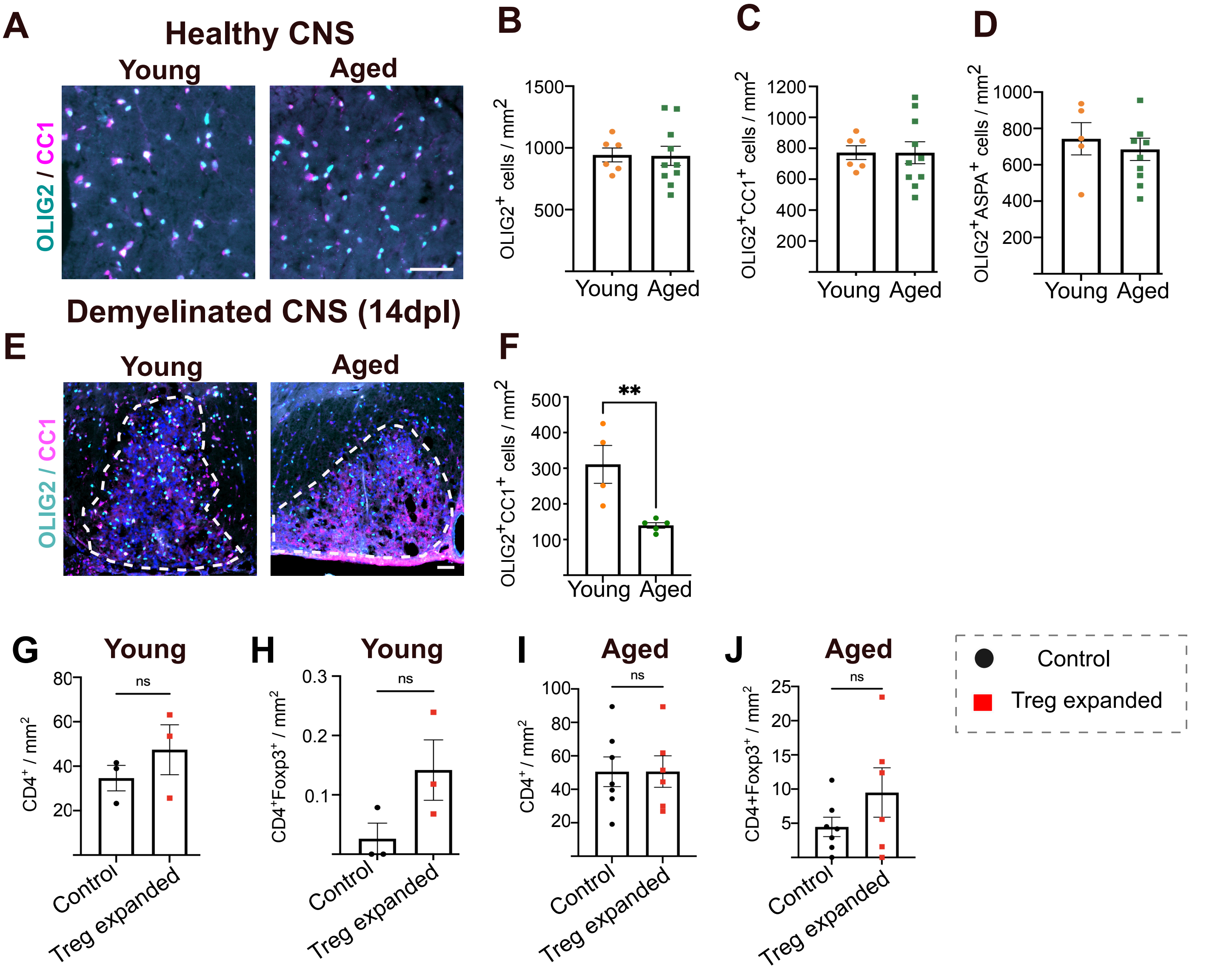
A
Healthy CNS
Young
Aged
OLIG2 / CC1
B
OLIG2+ cells / mm2
Young Aged
C
OLIG2+CC1+ cells / mm2
Young Aged
D
OLIG2+ASPA+ cells / mm2
Young Aged
Demyelinated CNS (14dpl)
E
Young
Aged
OLIG2 / CC1
F
OLIG2+CC1+ cells / mm2
**
Young Aged
G
Young
CD4+ / mm2
ns
Control
Treg expanded
H
Young
CD4+Foxp3+ / mm2
ns
Control
Treg expanded
I
Aged
CD4+ / mm2
ns
Control
Treg expanded
J
Aged
CD4+Foxp3+ / mm2
ns
Control
Treg expanded
Control
Treg expanded

Sup. Fig. 2

Supplementary Figure 2: OPC differentiation is impaired in the aged CNS. A Representative images of OLIG2 (cyan) and CC1 (magenta) in the healthy white matter of young and aged mice (scale bar = 50 µm). **B** Bar graph quantification showing OLIG2$^+$ oligodendrocyte lineage cell density in young (n=6 mice) and aged (n=10 mice) healthy white matter (unpaired two-tailed Student's t-test, t=0.071, P=0.9437). **C** Bar graph quantification showing OLIG2$^+$CC1$^+$ oligodendrocyte cell density in young (n=6 mice) and aged (n=10 mice) healthy white matter (unpaired two-tailed Student's t-test, t=0.006, P=0.9949). **D** Bar graph quantification showing OLIG2$^+$ASPA$^+$ oligodendrocyte cell density in young (n=5 mice) and aged (n=9 mice) healthy white matter (unpaired two-tailed Student's t-test, t=0.960, P=0.3558). **E** Representative image of OLIG2 (cyan) and CC1 (magenta) immunostaining in lysolecithin-induced demyelinated white matter of young and aged control mice (scale bar = 50 µm, lesion is demarcated by the white dotted line). **F** Bar graph quantification showing OLIG2$^+$CC1$^+$ oligodendrocyte density in the demyelinated lesion area in young (n=4 mice) and aged (n=5 mice) control mice (unpaired two-tailed Student t-test, t=3.600, **P=0.0087). **G** Bar graph quantification showing the density of CD4$^+$ T cells in the demyelinated area of young control and Treg expanded mice (n=3 mice each, unpaired two-tailed Student's t-test, t=1.015, P=0.3673). **H** Bar graph quantification showing the density of CD4$^+$Foxp3$^+$ Treg in the demyelinated area of young control and Treg expanded mice (n=3 mice each, unpaired two-tailed Student's t-test, t=2.019, P=0.1137). **I** Bar graph quantification showing the density of CD4$^+$ T cells in the demyelinated area of aged control and Treg expanded mice (n=7 mice (control) and n=6 mice (Treg expanded), unpaired two-tailed Student's t-test, t=0.011, P=0.9912). **J** Bar graph quantification showing the density of CD4$^+$Foxp3$^+$ Treg in the demyelinated area of aged control and Treg expanded mice (n=7 mice (control) and n=6 mice (Treg expanded), unpaired two-tailed Student's t-test, t=1.376, P=0.1961). Data are represented as mean ± SEM. Source data are provided as a Source Data File.

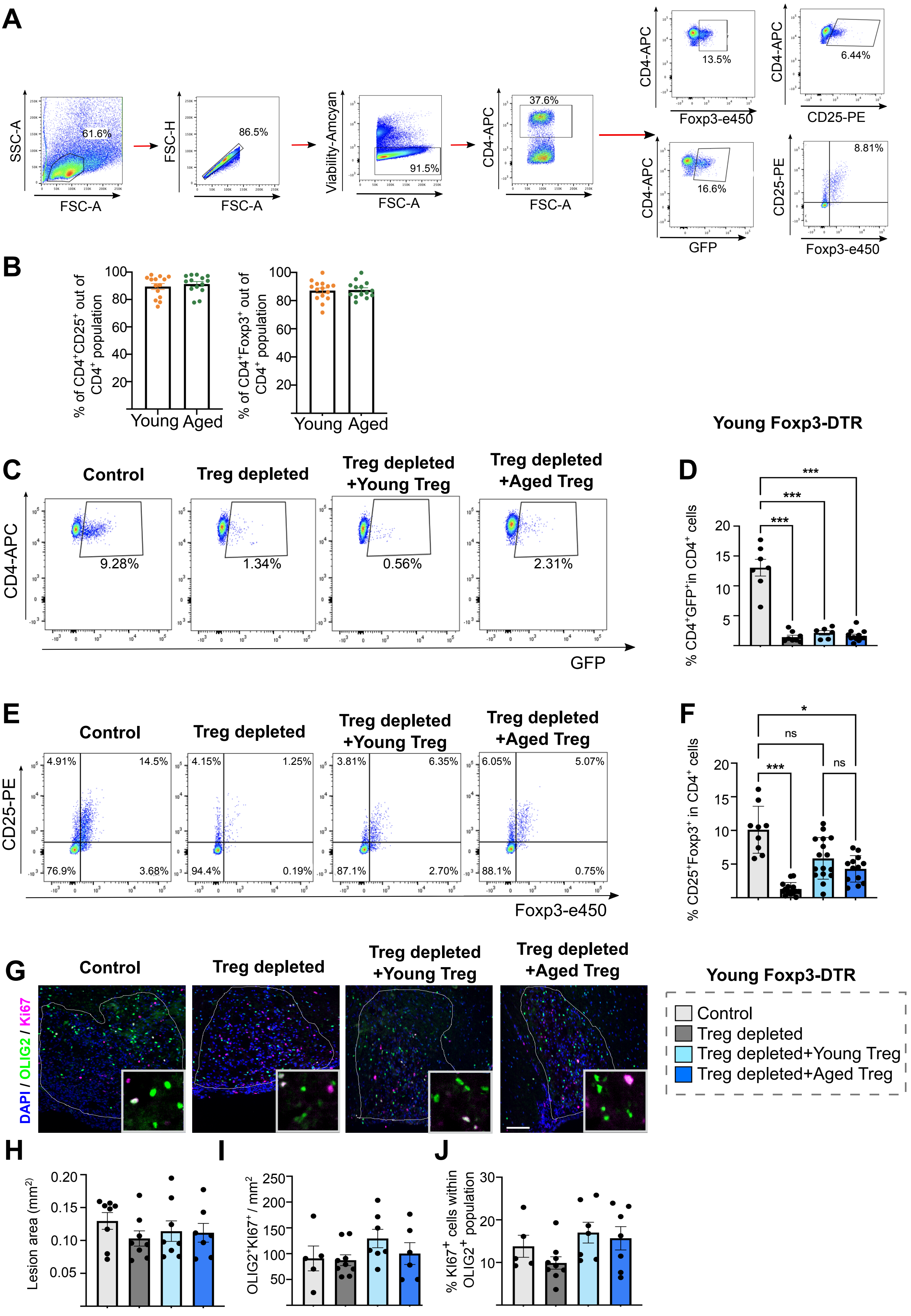
A
SSC-A
FSC-A
61.6%
FSC-H
86.5%
Viability-Amcyan
91.5%
CD4-APC
37.6%
13.5%
Foxp3-e450
6.44%
CD25-PE
16.6%
GFP
8.81%
B
% of CD4+CD25+ out of CD4+ population
% of CD4+Foxp3+ out of CD4+ population
Young
Aged
Young Foxp3-DTR
C
Control
Treg depleted
Treg depleted +Young Treg
Treg depleted +Aged Treg
9.28%
1.34%
0.56%
2.31%
D
% CD4+GFP+in CD4+ cells
***
E
4.91%
14.5%
76.9%
3.68%
4.15%
1.25%
94.4%
0.19%
3.81%
6.35%
87.1%
2.70%
6.05%
5.07%
88.1%
0.75%
Foxp3-e450
F
% CD25+Foxp3+ in CD4+ cells
*
ns
G
DAPI / OLIG2 / Ki67
Control
Treg depleted
Treg depleted+Young Treg
Treg depleted+Aged Treg
H
Lesion area (mm2)
I
OLIG2+KI67+ / mm2
J
% KI67+ cells within OLIG2+ population

Sup. Fig. 3

Supplementary Figure 3: Endogenous Treg depletion and reconstitution during lysolecithin- induced demyelination *in vivo.* **A** Gating strategy followed to identify natural Treg. **B** Bar graph showing natural Treg isolation purity for *in vitro* experiments and *in vivo* adoptive transfer studies. Natural Treg are identified by CD4 and CD25 expression (average 91% of the isolated cells for both young (n=13 isolations) and aged (n=14 isolations) are $CD4^{+}CD25^{+}$) and by the expression of CD4 and Foxp3 (average 87% of the isolated cells are positive for CD4 and Foxp3 in both young (n=14 isolations) and aged (n=15 isolations) mice (n=13-15 independent isolations, each isolation includes a pool comprised of 2 to 8 different mice, unpaired two-tailed Student's t test, after *arcsin* conversion, $t_{CD4+CD25+}$=0.996, $P_{CD4+CD25+}$=0.3284, $t_{CD4+Foxp3+}$=0.093, $P_{CD4+Foxp3+}$=0.9264). **C** Flow cytometric plot showing CD4 and GFP expression to identify endogenous natural Treg in lymph nodes. **D** Bar graph showing quantification for the proportion of endogenous GFP^{+} Treg within the $CD4^{+}$ T cell population in the lymph nodes of young Foxp3-DTR mice at 14dpl (n=7 mice (control), n=8 mice (Treg depleted), n=6 mice (Treg depleted+ young) n=12 mice (Treg depleted + aged), 1-way ANOVA, after *arcsin* conversion, Sidak's multiple comparisons test, F=67.64, ***$P_{control\ vs\ Treg\ depleted}$<0.0001, ***$P_{control\ vs\ young}$<0.0001, ***$P_{control\ vs\ aged}$<0.0001). **E** Flow cytometric plots showing CD25 and Foxp3 expression to detect Treg reconstitution in lymph nodes after adoptive transfer. **F** Bar graph quantification shows the proportion of $CD25^{+}Foxp3^{+}$ cells within the $CD4^{+}$ T cell population present in the lymph nodes of young Foxp3-DTR mice at 14dpl (n=9 mice (control), n=14 mice (Treg depleted), n=16 mice (Treg depleted +young), n=13 mice (Treg depleted+aged), Kruskal Wallis and Dunn's multiple comparisons test, KW statistic=31.92, ***$P_{control\ vs\ Treg\ depleted}$<0.0001, $P_{control\ vs\ young}$=0.1766, *$P_{control\ vs\ aged}$=0.0227, $P_{young\ vs\ aged}$>0.9999). **G** Representative images of immunostaining showing proliferating OPCs at 14 dpl (OLIG2 (green) and Ki67 (magenta)) (scale bar = 100 µm, demyelinated area is indicated by the white line). **H** Bar graph showing the area of demyelination in response to damage (n=8 mice (control), n=8 mice (Treg depleted, n=8 mice (Treg depleted+ young), n=7 mice (Treg depleted +aged), 1-way ANOVA, Sidak's multiple test correction, F=0.6835 , $P_{control\ vs\ Treg\ depleted}$=0.5286, $P_{control\ vs\ young}$=0.8857, $P_{control\ vs\ aged}$=0.8402, Pyoung vs aged>0.9999). **I** Bar graph showing the density of proliferating OPCs in the demyelinated area in response to damage (n=5 mice (control), n=9 mice (Treg depleted), n=7 mice (Treg depleted+ young), n=6 mice (Treg depleted +aged), 1-way ANOVA, Sidak's multiple test correction, F=1.302 , $P_{control\ vs\ Treg\ depleted}$=0.9999, $P_{control\ vs\ young}$=0.4894, $P_{control\ vs\ aged}$=0.9948, $P_{young\ vs\ aged}$=0.6898). **J** Bar graph showing the proportion of proliferating OPCs within oligodendrocyte lineage cells in the demyelinated area in response to damage (n=5 mice (control), n=9 mice (Treg depleted), n=7 mice (Treg depleted+ young), n=6 mice (Treg depleted +aged), 1-way ANOVA, Sidak's multiple test correction after arcsin conversion, F=1.354, $P_{control\ vs\ Treg\ depleted}$=0.7704, $P_{control\ vs\ young}$=0.9974, $P_{control\ vs\ aged}$=0.9509, $P_{young\ vs\ aged}$=0.9902). Data are represented as mean ± SEM. Source data are provided as a Source Data File.

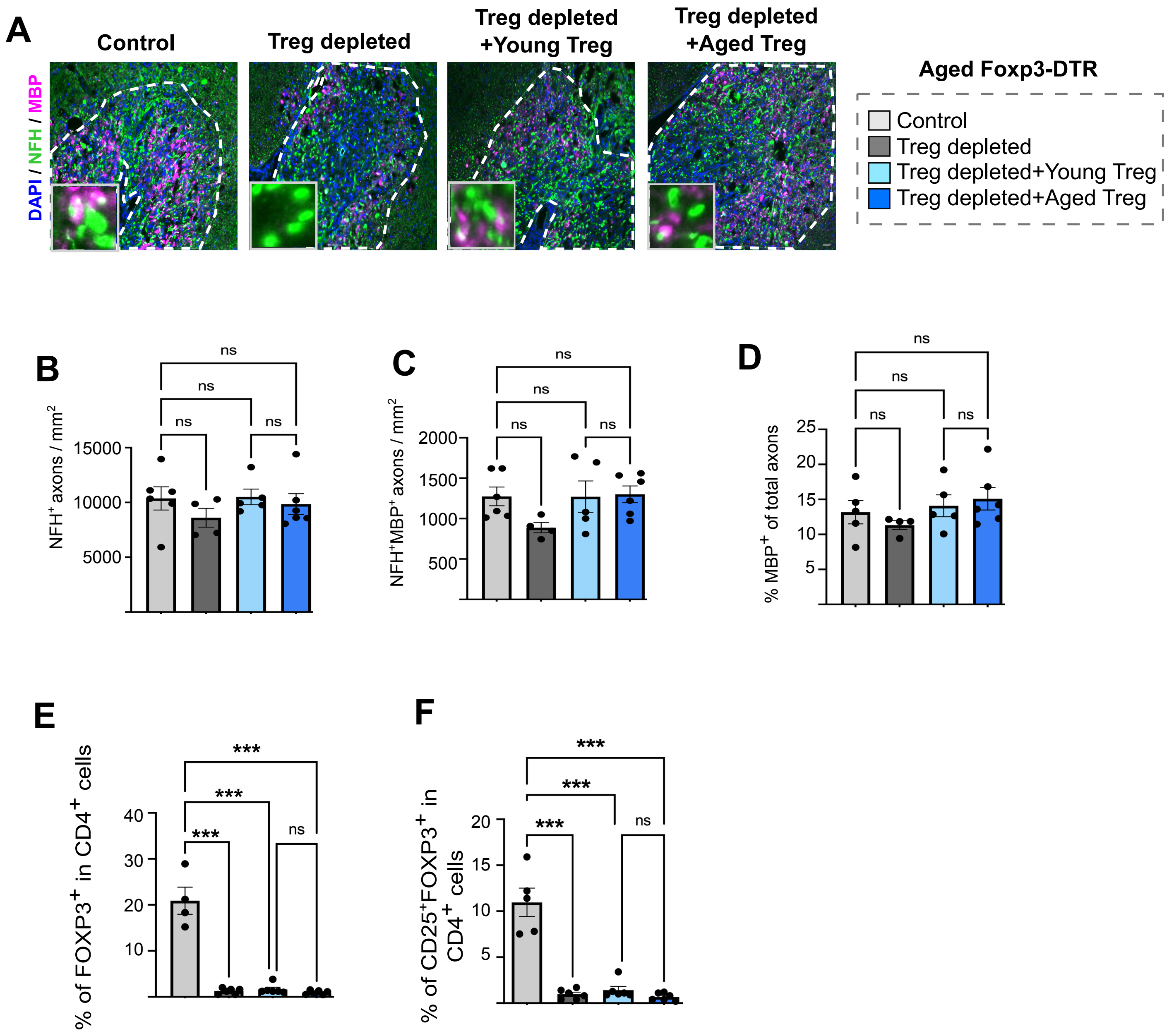
A
Control
Treg depleted
Treg depleted +Young Treg
Treg depleted +Aged Treg
DAPI / NFH / MBP
Aged Foxp3-DTR
Control
Treg depleted
Treg depleted+Young Treg
Treg depleted+Aged Treg
B
NFH+ axons / mm2
15000
10000
5000
ns
C
NFH+MBP+ axons / mm2
2000
1500
1000
500
ns
D
% MBP+ of total axons
25
20
15
10
5
ns
E
% of FOXP3+ in CD4+ cells
40
30
20
10
***
ns
F
% of CD25+FOXP3+ in CD4+ cells
20
15
10
5
***
ns

Sup. Fig. 4

Supplementary Figure. 4: Endogenous Treg depletion and reconstitution during lysolecithin- induced demyelination *in vivo* in aged mice. A Representative images of immunostaining for neurofilament-H (NFH, green) and MBP (magenta) to quantify myelin wrapping as an early indicator of remyelination at 14 dpl (scale bar = 50 µm, demyelination area is indicated by the white dotted line). **B** Quantification shows the total number of axons in the demyelinated area of aged mice (n=6 mice (control), n=4 mice (Treg depleted), n=5 mice (Treg depleted + young), n=6 mice (Treg depleted + aged), 1-way ANOVA, Sidak's multiple comparisons test, F=0.6998, $P_{\text{control vs Treg depleted}}$=0.6388, $P_{\text{control vs young}}$>0.9999, $P_{\text{control vs aged=}}$0.9901, $P_{\text{young vs aged}}$=0.9800). **C** Quantification shows the density of MBP-wrapped axons in the demyelinated area of aged mice (n=6 mice (control), n=4 mice (Treg depleted), n=5 mice (Treg depleted + young), n=6 mice (Treg depleted + aged), 1-way ANOVA, Sidak's multiple comparisons test, F=1.894, $P_{\text{PBS vs Treg depleted}}$=0.2229, $P_{\text{PBS vs young}}$>0.9999, $P_{\text{PBS vs aged}}$=0.9998, $P_{\text{young vs aged}}$=0.9998). **D** Quantification shows the percentage of MBP-wrapped axons in the demyelinated area of aged mice (n=6 mice (control), n=4 mice (Treg depleted), n=5 mice (Treg depleted + young), n=6 mice (Treg depleted + aged), Kruskal Wallis test, Dunn's multiple comparisons test, KW statistic=3.766, $P_{\text{PBS vs Treg depleted}}$>0.9999, $P_{\text{PBS vs young}}$>0.9999, $P_{\text{PBS vs aged}}$=>0.9999, $P_{\text{young vs aged}}$>0.9999). **E** Bar graph quantification showing reconstitution of $FOXP3^{+}$ Treg within the $CD4^{+}$ population in the lymph nodes of aged Foxp3-DTR at the point of sacrifice (n=4 mice (control), n=6 mice (Treg depleted), n=6 mice (Treg depleted + young), n=6 mice (Treg depleted + aged), 1-way ANOVA, Sidak's multiple comparisons test after arcsin conversion, F=65.69, ***$P_{\text{PBS vs Treg depleted}}$<0.0001, ***$P_{\text{PBS vs young}}$<0.0001, ***$P_{\text{PBS vs aged}}$<0.0001, $P_{\text{young vs aged}}$=0.9803). **F** Bar graph quantification showing the proportion of $CD25^{+}FOXP3^{+}$ Treg in $CD4^{+}$ T cells population in the lymph nodes of aged Foxp3-DTR at the point of sacrifice (n=5 mice (control), n=6 mice (Treg depleted), n=6 mice (Treg depleted + young), n=6 mice (Treg depleted + aged), 1-way ANOVA, Sidak's multiple comparisons test after *arcsin* conversion, F=44.5, ***$P_{\text{PBS vs Treg depleted}}$<0.0001, ***$P_{\text{PBS vs young}}$<0.0001, ***$P_{\text{PBS vs aged}}$<0.0001, $P_{\text{young vs aged}}$=0.9135). Data are represented as mean ± SEM. Source data are provided as a Source Data File.

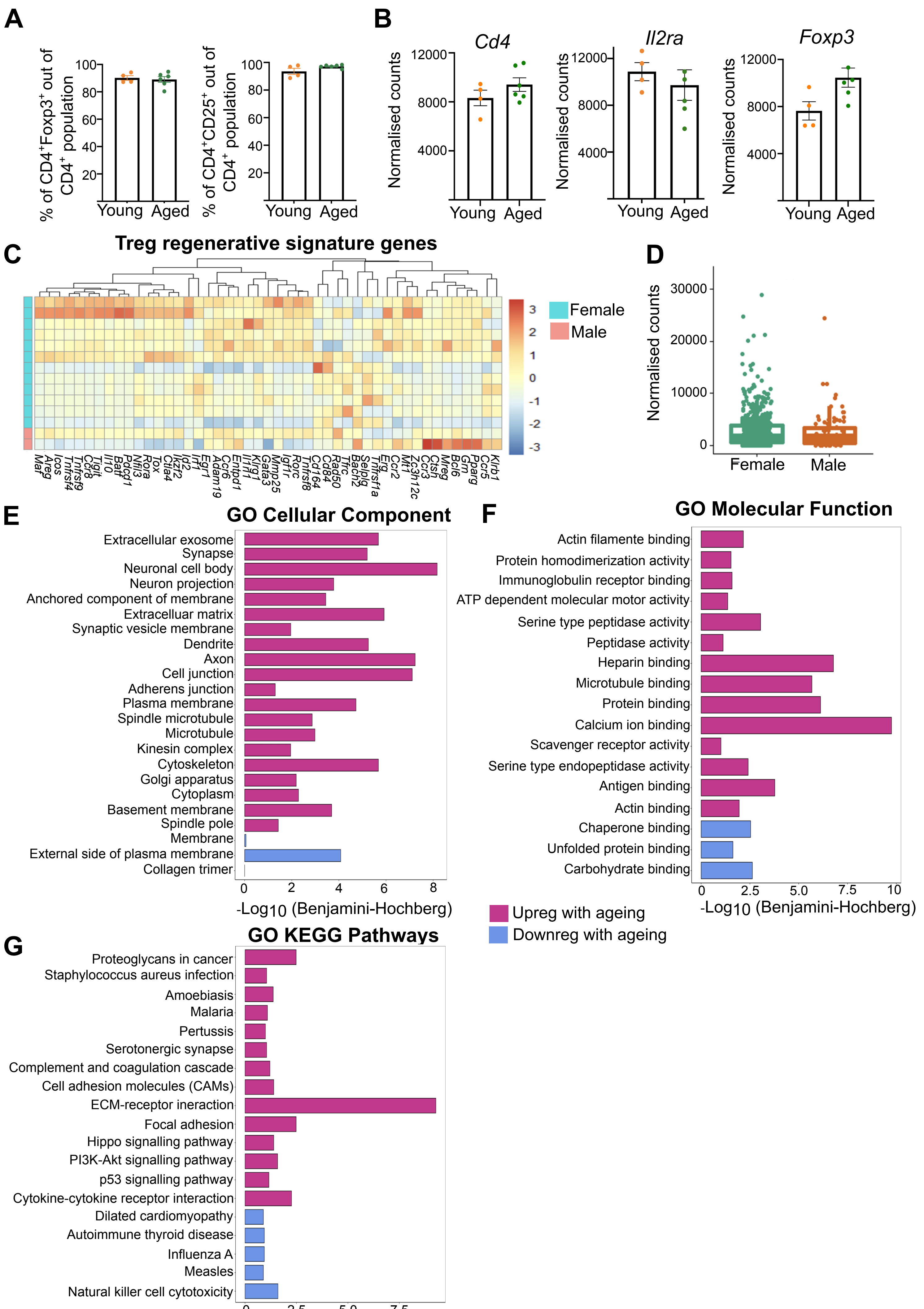

A
% of CD4+Foxp3+ out of CD4+ population
% of CD4+CD25+ out of CD4+ population
Young
Aged
B
Cd4
Il2ra
Foxp3
Normalised counts
C
Treg regenerative signature genes
Female
Male
Maf Areg Icos Tnfrsf4 Tnfrsf9 Ccr8 Tigit Il10 Batf Pdcd1 Nfil3 Rora Tox Ctla4 Ikzf2 Id2 Irf1 Egr1 Adam19 Ccr6 Entpd1 Il1rl1 Klrg1 Gata3 Mmp25 Igf1r Rorc Tnfrsf8 Cd164 Cd84 Rad50 Tfrc Bach2 Selplg Tnfrsf1a Erg Ccr2 Mt1 Zc3h12c Ccr3 Ctsh Mreg Bcl6 Gm Pparg Ccr5 Klrb1
D
Normalised counts
Female
Male
E
GO Cellular Component
Extracellular exosome
Synapse
Neuronal cell body
Neuron projection
Anchored component of membrane
Extracelluar matrix
Synaptic vesicle membrane
Dendrite
Axon
Cell junction
Adherens junction
Plasma membrane
Spindle microtubule
Microtubule
Kinesin complex
Cytoskeleton
Golgi apparatus
Cytoplasm
Basement membrane
Spindle pole
Membrane
External side of plasma membrane
Collagen trimer
-Log10 (Benjamini-Hochberg)
F
GO Molecular Function
Actin filamente binding
Protein homodimerization activity
Immunoglobulin receptor binding
ATP dependent molecular motor activity
Serine type peptidase activity
Peptidase activity
Heparin binding
Microtubule binding
Protein binding
Calcium ion binding
Scavenger receptor activity
Serine type endopeptidase activity
Antigen binding
Actin binding
Chaperone binding
Unfolded protein binding
Carbohydrate binding
-Log10 (Benjamini-Hochberg)
Upreg with ageing
Downreg with ageing
G
GO KEGG Pathways
Proteoglycans in cancer
Staphylococcus aureus infection
Amoebiasis
Malaria
Pertussis
Serotonergic synapse
Complement and coagulation cascade
Cell adhesion molecules (CAMs)
ECM-receptor ineraction
Focal adhesion
Hippo signalling pathway
PI3K-Akt signalling pathway
p53 signalling pathway
Cytokine-cytokine receptor interaction
Dilated cardiomyopathy
Autoimmune thyroid disease
Influenza A
Measles
Natural killer cell cytotoxicity
-Log10 (Benjamini-Hochberg)

Sup. Fig. 5

Supplementary Figure 5: Downstream analysis of differentially expressed genes between young and aged natural Treg. **A** Bar graphs showing the purity of young and aged natural Treg subjected to RNA sequencing (on average 89% of $CD4^+$ cells were $Foxp3^+$, while 94% of $CD4^+$ cells were $CD25^+$) (n=4 mice (young) and n=6 mice (aged), Mann-Whitney U test, $U_{CD4Foxp3}$=11, $P_{CD4Foxp3}$=0.9143, $U_{CD4CD25}$=6.5, $P_{CD4CD25}$=0.2810). **B** Bar graph showing normalised counts of natural Treg marker transcripts *Cd4, IL2ra* and *Foxp3* (n=4 mice young, n=6 mice aged, Wald test, Bonferroni multiple test correction, P_{Cd4}=0.4493, P_{Il2ra}=0.7045, P_{Foxp3}=0.0523). **C** Heatmap of normalised count values of the Treg tissue repair programme in male and female Treg. **D** Box plot showing the Treg tissue repair programme signature gene score of natural Treg of male and female mice (n=2 male mice, n=12 female mice, 47 genes associated to tissue repair Treg signature considered, two tailed unpaired Wilcoxon test, P= 0.63; Data shown as box plots in which the centre line denotes the median value (50th percentile), while the box contains the 25th to 75th percentile datasets). **E-G** Bar graphs showing the GO cellular component, molecular function and KEGG pathways enriched within the differentially expressed genes upregulated (magenta) and downregulated (purple) in aged Treg. Data are represented as mean ± SEM. Source data are provided as a Source Data File.

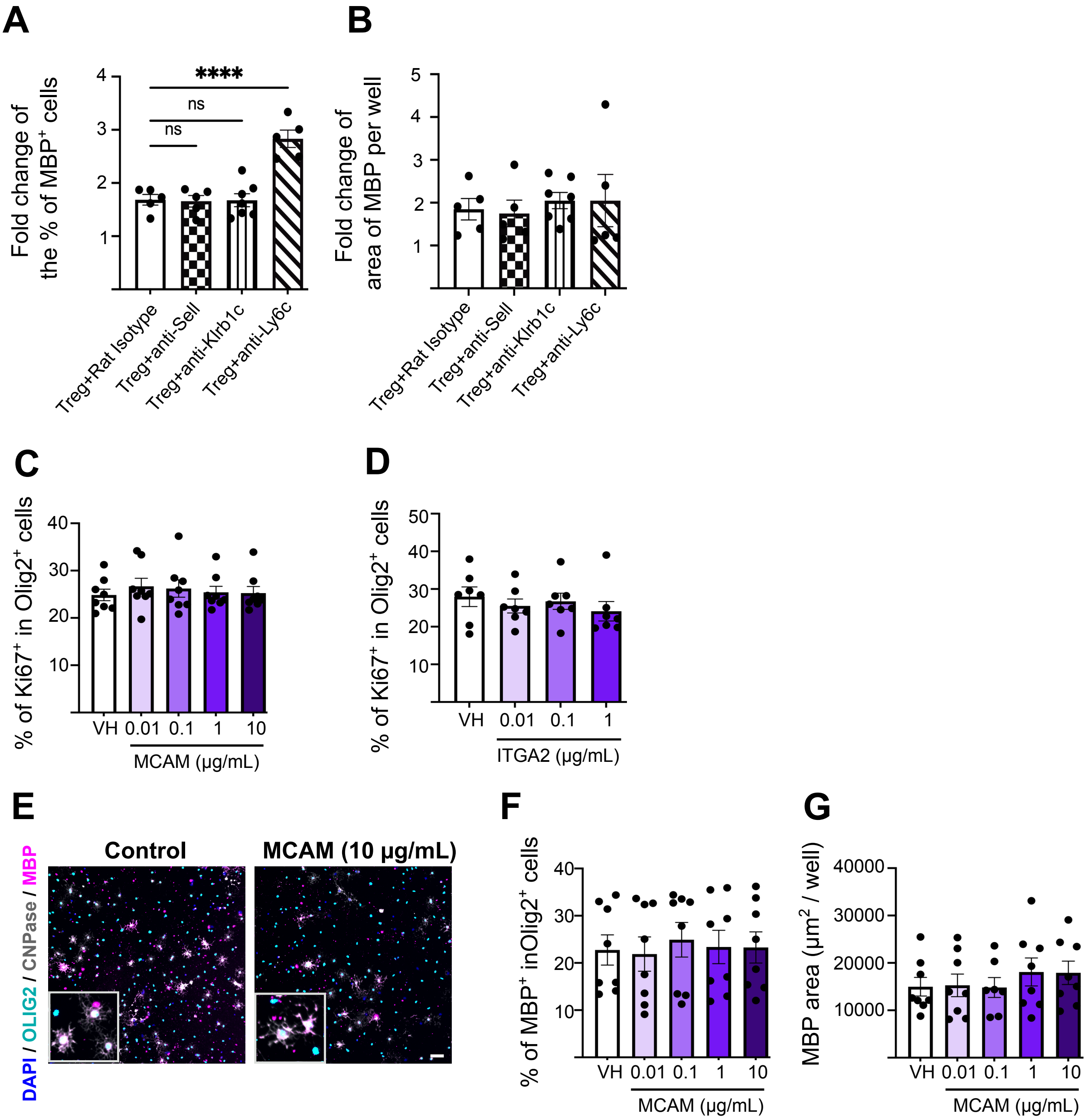
A
B
C
D
E
F
G
Fold change of the % of MBP+ cells
Fold change of area of MBP per well
****
ns
ns
Treg+Rat Isotype
Treg+anti-Sell
Treg+anti-Klrb1c
Treg+anti-Ly6c
% of Ki67+ in Olig2+ cells
VH
0.01
0.1
1
10
MCAM (µg/mL)
ITGA2 (µg/mL)
DAPI / OLIG2 / CNPase / MBP
Control
MCAM (10 µg/mL)
% of MBP+ inOlig2+ cells
MBP area (µm2 / well)

Sup. Fig. 6

Supplementary Figure 6: MCAM does not directly promote OPC differentiation. A Bar graph showing the quantification of OPC differentiation in OPC and young Treg co-cultures in the presence or absence of neutralising antibodies against candidate cell surface mediators, measured by the fold change in the percentage of MBP^+ cells (n=5 mice (Treg+rat isotype), n=5 mice (Treg+anti-Sell), n=7 mice (Treg+anti-Klrb1c), n=5 mice (Treg+anti-Ly6c1), 2 independent experiments, 2-way ANOVA, Dunnet's multiple comparisons test, $F_{raw\ factor(experiment)}$=2.271, $P_{raw\ (experiment)}$=0.1068, $F_{column\ (treatment)}$=26.69, ***$P_{column\ (treatment)}$<0.0001 ; $P_{Treg\ rat\ isotype\ vs\ Treg\ anti-sell}$=0.9958, $P_{Treg\ rat\ isotype\ vs\ Treg\ anti-Klrb1c}$=0.9993, ***$P_{Treg\ rat\ isotype\ vs\ Treg\ anti-Ly6c1}$<0.0001). **B** Bar graph showing the quantification of OPC differentiation in OPC and young Treg co-cultures in the presence or absence of neutralising antibodies against candidate cell surface mediators, measured by the fold change in MBP^+ area per well (n=5 mice (Treg+rat isotype), n=5 mice (Treg+anti-Sell), n=7 mice (Treg+anti-Klrb1c), n=5 mice (Treg+anti-Ly6c1), 2 independent experiments, 2-way ANOVA, Dunnet's multiple comparisons test, $F_{raw\ factor(experiment)}$=5.447, **$P_{raw\ (experiment)}$=0.0062, $F_{column\ (treatment)}$=0.2906, $P_{column\ (treatment)}$=0.8314; $P_{Treg\ rat\ isotype\ vs\ Treg\ anti-sell}$=0.9795, $P_{Treg\ rat\ isotype\ vs\ Treg\ anti-Klrb1c}$=0.9987, $P_{Treg\ rat\ isotype\ vs\ Treg\ anti-Ly6c1}$=0.8691). **C** Bar graph showing quantification of the proportion of proliferating OPC ($Ki67^+$ cells) in rMCAM-treated neonatal OPCS at 6 days *in vitro* (n=7 mice, 2 independent experiments, Kruskal Wallis, Dunn's multiple test correction, KW statistic=1.637, $P_{VH\ vs\ 0.01\mu g/mL\ MCAM}$>0.9999, $P_{VH\ vs\ 0.1\mu g/mL\ MCAM}$>0.9999, $P_{VH\ vs\ 1\mu g/mL\ MCAM}$>0.9999, $P_{VH\ vs\ 10\mu g/mL\ MCAM}$>0.9999). **D** Bar graph showing quantification of the proportion of proliferating OPC ($Ki67^+$ cells) in rITGA2-treated neonatal OPCS at 3 days *in vitro* (n=7 mice, 2 independent experiments, Kruskal Wallis test, Dunn's multiple comparison test, KW statistic=1.695, $P_{VH\ vs\ 0.01\mu g/mL\ ITGA2}$>0.9999, $P_{VH\ vs\ 0.1\mu g/mL\ ITGA2}$>0.9999, $P_{VH\ vs\ 1\mu g/mL\ ITGA2}$=0.6509). **E** Representative images of immunostaining showing OPC differentiation in neonatal OPCs treated with recombinant MCAM (OLIG2 (cyan), CNPase (grey) and MBP (magenta), scale bar = 50 µm). **F** Bar graph showing quantification of the proportion of MBP-expressing oligodendrocytes in rMCAM-treated neonatal OPCS at 6 days *in vitro* (n=7 mice, 2 independent experiments, Kruskal Wallis, Dunn's multiple test correction, KW statistic=0.5085, $P_{VH\ vs\ 0.01\mu g/mL\ MCAM}$>0.9999, $P_{VH\ vs\ 0.1\mu g/mL\ MCAM}$>0.9999, $P_{VH\ vs\ 1\mu g/mL\ MCAM}$>0.9999, $P_{VH\ vs\ 10\mu g/mL\ MCAM}$>0.9999). **G** Bar graph showing quantification of the total MBP area per well in rMCAM-treated neonatal OPCS at 6 days *in vitro* (n=7 mice, 2 independent experiments, 1-way ANOVA, Sidak's Multiple test correction, F=0.4634, $P_{VH\ vs\ 0.01\mu g/mL\ MCAM}$>0.9999, $P_{VH\ vs\ 0.1\mu g/mL\ MCAM}$>0.999 $P_{VH\ vs\ 1\mu g/mL\ MCAM}$=0.7656, $P_{VH\ vs\ 10\mu g/mL\ MCAM}$=0.8005;). Data are represented as mean ± SEM. Source data are provided as a Source Data File.

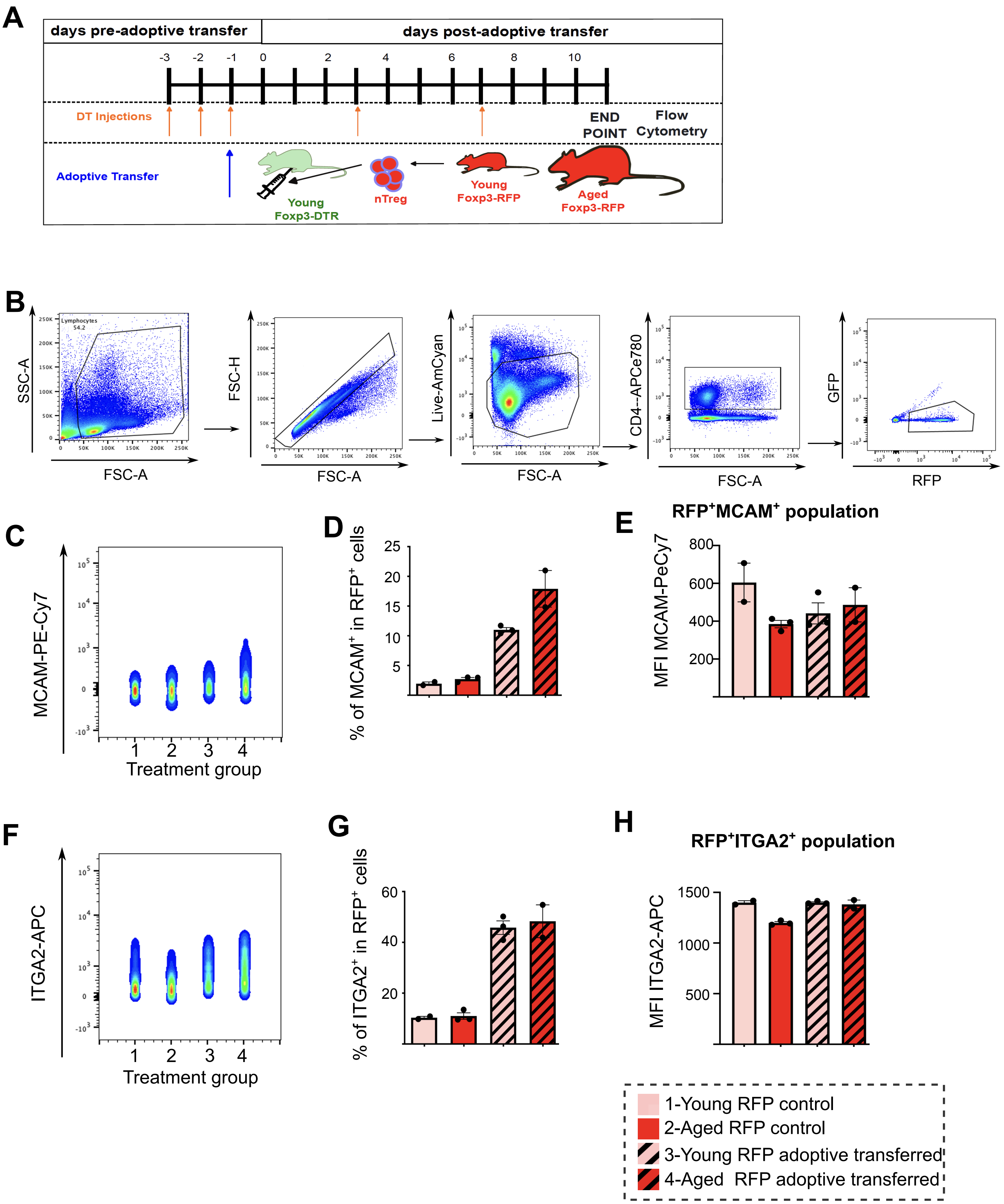
A
days pre-adoptive transfer
days post-adoptive transfer
DT Injections
END POINT
Flow Cytometry
Adoptive Transfer
Young Foxp3-DTR
nTreg
Young Foxp3-RFP
Aged Foxp3-RFP
B
SSC-A
FSC-A
Lymphocytes
FSC-H
Live-AmCyan
CD4--APCe780
GFP
RFP
C
MCAM-PE-Cy7
Treatment group
D
% of MCAM+ in RFP+ cells
E
RFP+MCAM+ population
MFI MCAM-PeCy7
F
ITGA2-APC
Treatment group
G
% of ITGA2+ in RFP+ cells
H
RFP+ITGA2+ population
MFI ITGA2-APC
1-Young RFP control
2-Aged RFP control
3-Young RFP adoptive transferred
4-Aged RFP adoptive transferred

Sup. Fig. 7

Supplementary Figure 7: MCAM and ITGA2 levels are restored in aged Treg upon adoptive transfer into young mice. A Diagram explaining the experimental design of *in vivo* Treg depletion and RFP$^+$ Treg adoptive transfer. **B** Gating strategy for flow cytometric analyses. **C** Flow cytometric plots showing MCAM expression in splenic RFP$^+$ Treg in control young and aged Foxp3-RFP mice and young Foxp3-DTR mice that received young or aged RFP$^+$ cells (data shows 670 cells from the concatenation of 2-3 mice in each). **D** Bar graph quantification of the proportion of MCAM$^+$ cells within RFP population (n=2 mice (young RFP control and aged RFP adoptive transferred), n=3 mice (aged RFP control and young RFP adoptive transferred), no statistical analysis performed). **E** Bar graph showing the median fluorescent intensity of MCAM in MCAM$^+$RFP$^+$ cells (n=2 mice (young RFP control and aged RFP adoptive transferred), n=3 mice (aged RFP control and young RFP adoptive transferred), no statistical analysis performed). **F** Flow cytometric plots showing ITGA2 expression in splenic RFP$^+$ Treg in control young and aged Foxp3-RFP mice and young Foxp3-DTR mice that received young or aged RFP$^+$ cells (data shows 670 cells from the concatenation of 2-3 mice in each). **G** Bar graph quantification of the proportion of ITGA2$^+$ cells within RFP population (n=2 mice (young RFP control and aged RFP adoptive transferred), n=3 mice (aged RFP control and young RFP adoptive transferred), no statistical analysis performed). **H** Bar graph showing the median fluorescent intensity of ITGA2 in ITGA2$^+$RFP$^+$ cells (n=2 mice (young RFP control and aged RFP adoptive transferred), n=3 mice (aged RFP control and young RFP adoptive transferred), no statistical analysis performed). Source data are provided as a Source Data File.
