# Supplementary material for: Ageing impairs the regenerative capacity of regulatory T cells in mouse central nervous system remyelination
Source: Nat Commun. 2024 Mar 11;15:1870. doi: 10.1038/s41467-024-45742-w (PMC10928230; doi:10.1038/s41467-024-45742-w)
Supplement: Supplementary file 3 — Description of Additional Supplementary Files [file 41467_2024_45742_MOESM3_ESM.docx]

**Supplementary Data 1:** Statistical analysis of the differentially expressed genes between young and aged Treg. Related to Fig. 6 and Sup. Fig. 5.
