# Supplementary material for: Ageing impairs the regenerative capacity of regulatory T cells in mouse central nervous system remyelination
Source: Nat Commun. 2024 Mar 11;15:1870. doi: 10.1038/s41467-024-45742-w (PMC10928230; doi:10.1038/s41467-024-45742-w)
Supplement: Supplementary file 5 — Reporting Summary [file 41467_2024_45742_MOESM5_ESM.pdf]

nature portfolio

Corresponding author(s): Prof. Denise Fitzgerald, Dr. Alerie Guzman de la Fuente

Last updated by author(s): Jan 4, 2024

Reporting Summary

Nature Portfolio wishes to improve the reproducibility of the work that we publish. This form provides structure for consistency and transparency in reporting. For further information on Nature Portfolio policies, see our Editorial Policies and the Editorial Policy Checklist.

Statistics

For all statistical analyses, confirm that the following items are present in the figure legend, table legend, main text, or Methods section.

n/a	Confirmed	
☐	☒	The exact sample size (*n*) for each experimental group/condition, given as a discrete number and unit of measurement
☐	☒	A statement on whether measurements were taken from distinct samples or whether the same sample was measured repeatedly
☐	☒	The statistical test(s) used AND whether they are one- or two-sided<br>*Only common tests should be described solely by name; describe more complex techniques in the Methods section.*
☒	☐	A description of all covariates tested
☐	☒	A description of any assumptions or corrections, such as tests of normality and adjustment for multiple comparisons
☐	☒	A full description of the statistical parameters including central tendency (e.g. means) or other basic estimates (e.g. regression coefficient) AND variation (e.g. standard deviation) or associated estimates of uncertainty (e.g. confidence intervals)
☐	☒	For null hypothesis testing, the test statistic (e.g. *F*, *t*, *r*) with confidence intervals, effect sizes, degrees of freedom and *P* value noted<br>*Give P values as exact values whenever suitable.*
☒	☐	For Bayesian analysis, information on the choice of priors and Markov chain Monte Carlo settings
☒	☐	For hierarchical and complex designs, identification of the appropriate level for tests and full reporting of outcomes
☒	☐	Estimates of effect sizes (e.g. Cohen's *d*, Pearson's *r*), indicating how they were calculated

Our web collection on statistics for biologists contains articles on many of the points above.

Software and code

Policy information about availability of computer code

Data collection	BD FACS Diva 9.0 was used to acquire most flow cytometry data except the young and aged brain and spinal cord flow cytometry data that was acquired with Cytek Aurora. Cell Insight CX5 HSC studio 6.6.1 imaging and analysis platform was used to acquire glial cell culture data and Leica LAS X 3.5.7.23225 and Leica Las X 3.9.0.28093 software was used to acquire brain slice and histology data. Transwell OPC data images were acquired with EVOS Fl Auto2 software (Fisher Scientific).
Data analysis	Images were processed and analysed in Fiji version v2.14.0, Cell Insight CX5 analysis platform HCS studio version v6.6.1, Cell Profiler v4.1.3, Cell Profiler Analyst v2.2.1 and blinded when manually counted. Statistical analysis was performed with Prism v9.0 or v10.0. RNA sequencing data was processed using FastQC for quality control, trimming was performed with Cut Adapt and mapped using STAR aligner (v 2.7.0a). Quantification was performed using featureCounts tool (v.2.0.0) and the read counts matrix analysed using DESeq2 (v1.38.1) R package in R Studio. Gene Ontology Enrichment and KEGG pathway analysis was done using DAVID webtool, as well as enrichGO and clusterProfiler (v.4.2) packages in R Studio. Protein-protein interaction was analysed using String software. Flow cytometry analysis was done with FlowJo version v9.0 and v10.0 or Spectroflo (Cytek).

For manuscripts utilizing custom algorithms or software that are central to the research but not yet described in published literature, software must be made available to editors and reviewers. We strongly encourage code deposition in a community repository (e.g. GitHub). See the Nature Portfolio guidelines for submitting code & software for further information.

Data

Policy information about availability of data

All manuscripts must include a data availability statement. This statement should provide the following information, where applicable:

- Accession codes, unique identifiers, or web links for publicly available datasets
- A description of any restrictions on data availability
- For clinical datasets or third party data, please ensure that the statement adheres to our policy

The manuscript has generated new RNA sequencing dataset showing young and aged Treg transcriptome which has been deposited to the Gene Expression Omnibus dataset (NCBI; accession number GSE218804) and is publicly available. Additionally, we have used for Figure 7 data from the following publicly available dataset (NCBI; accession number GSE52564). The rest of the data is available in the source data file uploaded with the manuscript as indicated in the data availability statement included in the manuscript.

Research involving human participants, their data, or biological material

Policy information about studies with human participants or human data. See also policy information about sex, gender (identity/presentation), and sexual orientation and race, ethnicity and racism.

Reporting on sex and gender	N/A
Reporting on race, ethnicity, or other socially relevant groupings	N/A
Population characteristics	N/A
Recruitment	N/A
Ethics oversight	N/A

Note that full information on the approval of the study protocol must also be provided in the manuscript.

Field-specific reporting

Please select the one below that is the best fit for your research. If you are not sure, read the appropriate sections before making your selection.

[x] Life sciences [ ] Behavioural & social sciences [ ] Ecological, evolutionary & environmental sciences

For a reference copy of the document with all sections, see nature.com/documents/nr-reporting-summary-flat.pdf

Life sciences study design

All studies must disclose on these points even when the disclosure is negative.

Sample size	Sample sizes were guided by previous published studies using the experimental models reported (e.g. Dombrowski et al., Nat Neuro 2017).
Data exclusions	Young Foxp3DTR mice were not sufficiently depleted of endogenous Treg (% of GFP+ out of CD4+>3% in lymph nodes, spleen or blood) or that were not reasonably reconstituted with adoptive transfer (% Foxp3+ out of CD4+<3% in lymph nodes) were excluded from the study except for aged Foxp3DTR mice, in which adoptively transferred Treg were not found as indicated in the manuscript. Additionally, mice in which the lysolecithin lesion was smaller than 0.045µm2 were excluded from the analysis as smaller lesions have a different remyelination profile with faster remyelination that could bias the results.
Replication	For in vitro glial cell culture experiments 2 or 3 independent experiments were undertaken with successful replication. Three independent experiments were undertaken for brain slice experiments, which show the same difference across groups althought the myelination index level was variable. For in vivo experiments IL2 expansion experiments were done in one batch for young and a separate batch for aged C57BL6/J mice. Foxp3-DTR lysolecithin lesions for young Foxp3DTR were performed across 4 different batches of surgeries due to the complexity of the experiments and all replications show the same result. For aged Foxp3DTR mice, lysolecithin surgeries were performed in one batch. Flow cytometry analysis of Treg depletion and reconstitution was performed with each batch of surgeries obtaining similar results. 3 and 14 dpl lysolecithin surgeries to identify Treg expression were performed in two batches with successful replication while EAE experiment was performed once. RFP adoptive transfer experiment was performed only once and young and aged brain and spinal cord flow cytometry experiment was also performed only once. For histology analysis, 2-3 separate spinal cord sections were imaged and analysed per animal. RNA sequencing experiment was performed only once, were the RNA extraction and sequencing of all the samples was performed simultaneously.
Randomization	For all in vivo experiments, mice were randomised to experimental groups before treatments commenced. For all in vitro experiments, 3-4 animals were done in each independent replicate and cells from each animal randomly distributed in the same plate. Then different treatments were allocate randomly by columns to each well of the plate. For young and aged flow cytometry experiments mice were segregated by age and otherwise randomly allocated in different cages.
Blinding	All processing was performed by a blinded and a non-blidned assessor. All quantifications were done blindly when manually counted or

alternatively, through automatic image analysis softwares such as Cell Insight CX5 or Cell Profiler.

Reporting for specific materials, systems and methods

We require information from authors about some types of materials, experimental systems and methods used in many studies. Here, indicate whether each material, system or method listed is relevant to your study. If you are not sure if a list item applies to your research, read the appropriate section before selecting a response.

Materials & experimental systems

n/a	Involved in the study
☐	☒ Antibodies
☒	☐ Eukaryotic cell lines
☒	☐ Palaeontology and archaeology
☐	☒ Animals and other organisms
☒	☐ Clinical data
☒	☐ Dual use research of concern
☒	☐ Plants

Methods

n/a	Involved in the study
☒	☐ ChIP-seq
☐	☒ Flow cytometry
☒	☐ MRI-based neuroimaging

Antibodies

Antibodies used

Primary antibodies:
Anti-mouse IL-2 (Bioxcell, Clone JES-1A12, Cat. No. BE0043)
Mouse anti-A2B5 (Millipore, Clone A2B5-105, Cat No MAB312)
anti-IgM-Microbeads (Miltenyi, Cat No 130-047-301)
Goat anti-Olig2 (Bio-techne, Polyclonal, Cat No AF2418)
Rabbit anti-Olig2 (Millipore, PolyClonal, Cat No AB9610)
Mouse anti-CNPase (Sigma-Aldrich, Clone 12, Cat No C5922)
Rat anti-MBP (Millipore, Polyclonal, Cat No MAB386)
Rabbit anti-Ki67 (Abcam, Clone Sp6, Cat No ab16667)
Rat-Isotype-IgG2aK-APC (eBioscience, Clone eBr2a, Cat No 17-4321-82)
Mouse anti-Klrb1c-PE (ebiosciences, Clone PK136, Cat No A18483)
Rat anti-CD62L-APC (eBioscience, Clone Mel14, Cat No 17-0621-83)
Rat anti-Ly6c1-APC (eBioscience, Clone RB6-BC5, Cat No 47-5931-82)
Rabbit IgG (Vektor Labs, polyclonal, Cat No I-1000-5)
Rabbit anti-Itga2 (Abcam, Clone EPR5788, Cat No ab133557)
Rabbit anti-MCAM (Abcam, Clone EPR3208, Cat No b75769)
Chicken anti-NFH (EncorBiotech, Polyclonal, Cat No CPCA-FN-H)
Rat CD4-APC (eBioscience, Clone RM4.5, Cat No 17-0042-82)
Rat anti-CD25-PE (eBioscience, Clone PC61.4, Cat No 25-0251-82)
Mouse anti-CC1 (Abcam, Polyclonal, Cat No ab15794)
Rabbit anti-ASPA (Millipore, Polyclonal, Cat No ABN1698)
Rabbit anti-NFH (Abcam, Polyclonal ,Cat No 8135)
Ra anti Foxp3-e450 (eBioscience, Clone FJK-16S, Cat No 48-5773-82)
Rabbit anti-NG2 (Millipore, Polyclonal, AB5320)
Rat anti-CD3 (eBioscience, Clone 17A2, Cat No 14-0032-82)
Rat anti-CD4 (Biolegend, Clone RM4.5, Cat No 100506)
Rabbit anti-Foxp3 (BioTechne, Polyclonal, Cat No MAB8214)
Rat anti-CD45-biotin (eBioscience, Clone 30-F11, Cat No 13-0451-82)
Rat anti-CD45-BUV835 (BD Biosciencies, Clone 30-F11, Cat No 565967)
Rat anti-CD40e450 (eBioscience, Clone GK1.5, Cat No 48-0041-82)
Rat anti-CD8 PE Fire640 (Biolegend, Clone 53-6.7, Cat No 100790)
Rat anti-CD62K-PECy7 (eBioscience, Clone Mel-14, Cat No 25-0621-82)
Rat anti-CD11b-PerCpCy5.5 (eBioscience, Clone M1/70, Cat No 45-0112-82)
Rat anti-CD44-BV510 (Biolegend, Clone IM7, Cat No 100344)
Rat anti-CD69-PE (eBioscience, Clone H1.2F3, Cat No, 12-0691-82)
Rat anti-CD25-AF488 (eBioscience, Clone PC6.1, Cat No 5309251-82)
Rat anti-CD19-SB780 (eBioscience, Clone ID3, Cat No 78-0193-82)
Rat anti-Foxp3-APC (eBioscience, Clone FJK 16S, Cat No 17-5773-82; Miltenyi Clone REA788, Cat No 130-111-601)
Rat anti-ITGA2-APC (eBioscience, Clone DX5, 17-5971, 82)
Rat anti-MCAM-PECy7 (Biolegend, Clone ME9f1, Cat No 134714)
Rat anti-CD4-APCe780 (eBiosciencem Clone GK1.5, Cat No 11-0041-82)

Secondary antibodies:
AF488-donkey anti-rabbit (Thermofischer Scientific Cat No A-21206)

AF568-donkey anti-rat (Abcam, Cat no ab175475)
AF647 donkey anti-rat (Abcam, Cat No ab150155)
AF755 donkey anti-goat (ThermoFisher Scientific, Cat No SA5-10091)
AF488 donkey anti-goat (ThermoFisher Scientific, Cat No A-11055)
AF568 donkey anti- mouse (ThermoFischer Scientific, Cat No A-10037)
AF647 donkey anti-rabbit (ThermoFisher Scientific, Cat No A-31573)
AF647 donkey anti-mouse (ThermoFisher Scientific, Cat No A-31571)
FITC donkey anti-chicken (Abcam, Cat No ab63507)

Validation

Rat anti- Mouse IL-2 is validated by Bioxcell for its use with mouse tissue and widely used in the literature (Marshall et al, 2015, J immunol)
Mouse anti-A2B5 is validated by Bioxcell for its use with mouse tissue and widely used in the literature (Segel et al., 2019, Nature)
Goat anti-Olig2 is validated by Biotechne for its use with mouse tissue and widely used in the literature (Osipovitch et al., 2019, Cell Stem Cell)
Rabbit anti-Olig2 is validated by Millipore for its use in mouse tissue and widely used in the literature (De la Fuente et al., 2015, JCB)
Mouse anto-CNPase is validated by Sigma-Aldrich for its use with mouse tissue and widely used in the literature (zhang et al., 2018, Mol Neurobiol)
Rat anti-MBP is validated by Millipore for its use with mouse tissue and widely used in the literature (Haines et al., 2015, Nat Neurosci)
Rabbit anti-Ki67 is validated by Millipore for its use with mouse tissue and widely used in the literature (Nacke et al., 2021, Nat Comms)
Mouse anto-Nk1.1-PE is validated by eBioscience for its use with mouse tissue and widely used in the literature (Bracy et al. 2000, Blood)
Rat anti-CD62L -APC is validated by eBioscience for its use in mouse tissue and widely used in the literature (Qi et al, Nat Comms, 2022)
Rat anti-Ly6c1-APC is validated by eBioscience for its use with mouse tissue and widely used in the literature (Watanabe et al, 2020, Nat Comms)
Rabbit anti-MCAM is validated by Abcam for its use with mouse tissue and extensively used in the literature (Brechbucl HM et al, 2020, JCI Insight)
Rabbit anti-Itga2 is validated by Abcam for its use with mouse tissue and extensively used in the literature (Flemming et al, 2020, Mol Biol Cell)
Chicken anti-NFH is validated by Encorbio for its use with mouse tissue and extensiveley used in the literature (De la Vega-Gallardo et al, 2020, PNAS)
Rabbit anti-NFH is validated by Abcam for its use with mouse tissue and extensively used in the literature (Rivera et al, 2019, Glia)
Rat anti-CD4 (Clone RM4.5)APC is validated by eBioscience and is extensively used in the literature (oei et al, 2020, Cell Stem Cell)
Rat anti-CD25-PE (Clone PC6.1) is validated by eBioscience for its use with mouse tissue and extensively used in the literature (Cannons et al., 2021, Cell Reports)
Mouse anti-APC (Clone CC1) is validated by Abcam for its used with mouse tissue and extensively used in the literature (Brooks et al., 2021, Nat Comms)
Rabbit anti-ASPA is validated by Millipore for its use with mouse tissue and widely used in the literature (Pan et al, 2020, Nat Neuro)
Rabbit anti-NFH is validated by Abcam for its use with mouse tissue and extensively used in the literature (Mughrabi et al. 2021, Elife)
Rat anti Foxp3-e450 is validated by eBioscience for its use with mouse tissue and extensively use din the literature (Dombrowski et al, 2017, Nat Neuro)
Rabbit anti-NG2 is validated by Millipore for its use with mouse tissue and widely used in the literature (Orduz et al, 2015, eLIfe)
Rat anti-CD3 is validated by eBioscience for its use with mouse tissue and widely used in the literature (Gauta et al, 2020, J Neuroinflamamtion)
Rat anti CD45 is validated by eBioscience and Biolegend for its use with mouse tissue and is widely used in the literature (Pasciuto et al, 2020, Cell, Yshii et al, 2022, Nat Immunology).
Rat anti CD4 is validated by eBioscience for its use with mouse tissue and is widely used in the literature (Pasciuto et al, 2020, Cell, Yshii et al, 2022, Nat Immunology).
Rat anti CD8 is validated by Biolegend for its use with mouse tissue and is widely used in the literature (Pasciuto et al, 2020, Cell, Yshii et al, 2022, Nat Immunology).
Rat anti CD62L is validated by eBioscience for its use with mouse tissue and is widely used in the literature (Pasciuto et al, 2020, Cell, Yshii et al, 2022, Nat Immunology).
Rat anti CD1b is validated by eBioscience for its use with mouse tissue and is widely used in the literature (Pasciuto et al, 2020, Cell, Yshii et al, 2022, Nat Immunology).
Rat anti CD44 is validated by Biolegend for its use with mouse tissue and is widely used in the literature (Pasciuto et al, 2020, Cell, Yshii et al, 2022, Nat Immunology).
Rat anti CD69 is validated by eBioscience for its use with mouse tissue and is widely used in the literature (Pasciuto et al, 2020, Cell, Yshii et al, 2022, Nat Immunology).
Rat anti CD19 is validated by eBioscience for its use with mouse tissue and is widely used in the literature (Pasciuto et al, 2020, Cell, Yshii et al, 2022, Nat Immunology).
Rat anti CD25 is validated by eBioscience for its use with mouse tissue and is widely used in the literature (Pasciuto et al, 2020, Cell, Yshii et al, 2022, Nat Immunology).
Rabbit Foxp3 is validated by BioTechne for its use with mouse tissue and is widely used in the literature (Pasciuto et al, 2020, Cell)
Rat CD4 is validated by Biolegend for its use with mouse tissue and is widely used in the literature (Pasciuto et al, 2020, Cell)
Rat anti-ITGA2 is validated by eBioscience for its use with mouse tissue and widely used in the literature (Conde et al, 2021, Ncomms)
Rat anti-MCAM is validated by Biolegend for its use with mouse tissue and widely used in the literature (Grol et al., 2021, Elife)

Animals and other research organisms

Policy information about studies involving animals; ARRIVE guidelines recommended for reporting animal research, and Sex and Gender in Research

Laboratory animals	All animals included in this study are either C57BL6/J, Foxp3-DTR mice (Foxp3tm3(DTR/GFP)Ayr/J) or Foxp3-RFP (C57BL/6-Foxp3tm1Flv/J Foxp3-IRES-mRFP) bread in C57BL6/J background. Neonatal mice for glial cell cutlures were P3-P7 for brain slices were P3, while young mice ranged from 2-4m and aged mice were 15-18months when used for adoptive transfer, 16-22 months in the case of the ones used for the RNA sequencing and 19-23 months in the case of aged Foxp3-DTR.<br>Animals were housed under standard laboratory conditions 12/12h light/dark cycle at 23 degrees celsius, 46% humidity and food and water available ad libitum.
Wild animals	We did not used wild animals in this study.
Reporting on sex	Neonatal mice were mixed sexes. Adult C57BL6/J mice used for in vitro experiments were only male mice to avoid -Y chromosome crossreactivity when in contact with mixed sex neonatal OPCs or brain slices. For adoptive transfer experiments, donor C57BL6/J mice were females to avoid X-Y chromosome reactivity since the recipient young and aged Foxp3DTR mice were mixed gender. For RFP based adoptive transfer experiments, due to the limited availability of female Foxp3-RFP mice, the donors were mixed gender and they were paired with recipients of the same gender to avoid X-Y crossreactivity.
Field-collected samples	We did not used field-collected samples in this study.
Ethics oversight	Queen's University Belfast's Animal Welfare and Ethics Review Committee (PPL 2789, PPL 2894)

Note that full information on the approval of the study protocol must also be provided in the manuscript.

Plants

Seed stocks	We did not use plants in this study
Novel plant genotypes	We did not use plants in this study
Authentication	We did not use plants in this study

Flow Cytometry

Plots

Confirm that:

- [x] The axis labels state the marker and fluorochrome used (e.g. CD4-FITC).
- [x] The axis scales are clearly visible. Include numbers along axes only for bottom left plot of group (a 'group' is an analysis of identical markers).
- [x] All plots are contour plots with outliers or pseudocolor plots.
- [x] A numerical value for number of cells or percentage (with statistics) is provided.

Methodology

Sample preparation	Spleen and lymph nodes were mashed through a 70µm strainer. In the case of splenocytes, cells were exposed to red blood cell lysis buffer fro 2min at room temperature. Both, lymph nodes and splenocytes were then washed with PBS and centrifuged at 300g for 5min at 4 degrees Celsius. Cells were resuspended in 200mL PBS and stained for cell viability and cell surface staining with antibodies against CD4 (1:500, Clone RM4.5) and CD25 (1:500, Clone PC6.1) for 15min at room temperature. Cells were washed with flow cytometry staining buffer (FCSB) (2% FCS in PBS) and centrifuged at 300g for 5 min at 4 degrees Celsius. The pellet was resuspended in PBS and data acquired on a FACS Canto II with BD FACS Diva v9.0 software. Presence of endogenous nTreg was determined by the expression of GFP. To determine either purity of nTreg isolations of the extent of reconstitution of nTregs by adoptive transfer, cells were washed and centrifuged for 5 min at 300g at 4 degrees Celsius. Cells were then resuspended in 100µL Fix & Perm B with anti-Foxp3 (1:100, Clone FJK-16s) antibody overnight at 4 degrees Celsius. Cells were then washed with FCSB and centrifuged at 300g for 5 min at 4 degrees Celsius. Cells were then resuspended, data acquired on a FACS Canto II with BD FACS Diva v9,0 and were analysed with FlowJo v9.0 or v10.0 (BD). In the case of blood, to evaluate young and aged nTreg numbers, 30µL of blood were incubated with 100µL of the cell surface antibody mix for 30 min at room temperature. Cells were then washed with FSCB and centrifuged at 300g for 5min at 4 degrees Celsius. Cells were resuspended, fixed , and lysed in 100µL of Optilyse B for 10 min at room temperature. Cells were washed with distilled water, and after 15min data were acquired on a FACS Canto II with BD FACS Diva v9.0. After

initial data acquisition for GFP, remaining cells were centrifuged at 300g for 5min at 4 degrees Celsius and incubated overnight with anti-Foxp3 antibody in Fix & Perm buffer B, as described above.

For Brain and Spinal cord flow cytometry mice were injected i.v. with biotinylated CD45 antibody (30-F11, Bioscience) 3 minutes prior to euthanasia via a schedule 1 method. Brain, spinal cord and spleen tissues were dissected out and placed individually into 5mL cold FACS buffer (2% FCS in PBS). Single cell suspensions were prepared from brain and spinal cord tissues as previously described (Ref 60). Briefly, tissues were chopped into small pieces and digested for 30 min at 37°C with 0.4 mg/mL collagenase D (Sigma-Aldrich), 300 µg/mL hyaluronidase (Sigma-Aldrich) and 40 µg/mL DNase 1 (Sigma-Aldrich) in IMDM supplemented with 2 mM MgCl2, 2 mM CaCl2, 2 mM l-Glutamine (Sigma-Aldrich), 1 mM Sodium Pyruvate (Gibco) 10 mM HEPES (Gibco), 20% FBS and 1X Gentamycin (Abcam), followed by filtration (through 100 µm mesh) and enrichment for leukocytes by gradient centrifugation (40% Percoll GE healthcare, 600 x g, 15 min). To prepare single cell suspensions from spleen tissues, the spleen was mechanically disrupted between two glass slides, filtered through 100 µm mesh and red blood cells were lysed. Splenocytes were counted using a Countess automated cell counter. Following cell preparation, non-specific binding was blocked using 2.4G2 supernatant. Initial staining included eBioscience Fixable Viability Dye e780 (1:4000, eBioscience) and a fluorescence-tagged streptavidin to identify leukocytes which were from contaminating blood using the injected CD45-biotin (1:1000, eBioscience, Clone 30-F11). Next, a 1h surface stain was performed including antibodies for CD45 (1:500; BD Bioscience, Clone 30-F11), CD4 (1:500; eBioscience, Clone GK1.5), CD8 (1:1000 Biolegend, Clone 53-6.7), CD62L (1:1000; eBioscience, Clone Mel-14), CD11b (1:500; eBioscience, Clone M1/70), CD44 (1:500; Biolegend, Clone IM7), CD69 (1:100, eBioscience, Clone H1.2F3), CD25 (1:200; eBioscience, Clone PC61) and CD19 (1:250, eBioscience, Clone ID3) Subsequently, cells were fixed and permeabilised with the eBioscience Foxp3 staining kit (eBioscience) and a 1h intracellular stain for Foxp3 was added (1:100, eBioscience, Clone FJK-16s; Miltenyi, Clone REA788). For brain and spinal cord, the entire sample was acquired. Flow cytometry data were acquired on a Cytek Aurora and data collection performed using SpectroFlo (Cytek).

Instrument	BD FACS Canto II or Aurora Cytek
Software	BD FACS Diva version 9.0 for acquisitio and Flowjo v9,0 and v10.0 or Spetroflo (Cytek) for analysis.
Cell population abundance	Foxp3+ Tregs are about 2% of total lymph node, splee or blood and about 5-15% of the CD4+ T cell population.
Gating strategy	Gating was done on negatively stained controls, Fluorescence minus one controls and isotype controls and after single color stained laser compensation. To gate our populations we followed the subsequent steps to identify Tregs: 1) gate on FSC-A vs SSC-A, 2) gate on FSC-A vs FSC-H, 3) Gate on FSC-A vs Viability dye, 4) Gate on CD4+ population vs FSC-A, 5) Gate either on CD4 vs CD24 or CD4 vs Foxp3 or CD4 vs GFP or CD25 vs Fopx3 depending on the dataset.<br>For young and aged CNS flow cytometry characterisation we followed the subsequent steps: 1) gate on FSC-A vs SSC-A, 2) gate on FSC-A vs FSC-H, 3) Gate on FSC-A vs Viability dye, 4) Gate on CD45+ population vs FSC-A, 5) Gate on CD4 vs CD8, 6) Gate either on CD4 vs CD24 or CD4 vs Foxp3 or CD4 vs GFP or CD25 vs Fopx3 depending on the dataset and 7) Gated on CD69 + vs Foxp3.<br>For the RFP based Treg reconstitution experiments we followed the subsequent steps: 1) gate on FSC-A vs SSC-A, 2) gate on FSC-A vs FSC-H, 3) Gate on FSC-A vs Viability dye, 4) Gate on CD4+ population vs FSC-A, 5) Gate on RFP+ GFP- celsl and 6) Gate on ITGA2+ or MCAM+ and then measure median fluorescent intensity. This values were used for the bra graphs. For the flow plot shown in the figure we concatenated the same number of RFP+ cells in each group and then evaluated median fluorescent intensity of RFP+ITGA2+ or RFP+MCAM+ cells.

☒ Tick this box to confirm that a figure exemplifying the gating strategy is provided in the Supplementary Information.
